# Vocational challenges in congenital heart disease

**DOI:** 10.1007/s12471-014-0545-9

**Published:** 2014-04-01

**Authors:** W. A. Helbing, E. Utens

**Affiliations:** 1 Department of Pediatrics, Division of Cardiology, Sp-2429, ErasmusMC-Sophia Children’s Hospital, PO Box 2060, 3000 CB Rotterdam, the Netherlands; 2 Department of Child and Adolescent Psychiatry, KP-2 2865, ErasmusMC-Sophia Children’s Hospital, PO Box 2060, 3000 CB Rotterdam, the Netherlands

Despite advances in prenatal detection and counselling, congenital heart disease remains the most common type of congenital disease. In the last decades, treatment results in children have shown spectacular improvements, even in the most complex types of congenital heart disease. At present, the overall mortality rate for paediatric congenital heart surgery in the Netherlands is <2 %. The decline in mortality from this type of disease has resulted in an increasing number of adults with congenital heart disease. The national database for adult congenital heart disease, ConCor, currently has 14,860 people registered and it is estimated that many thousands of these patients have been lost to follow-up.

Recent research has shown that older children and young adults who have been treated for congenital heart disease are generally in good clinical condition. For patients treated with recent modifications of common treatment strategies, it has been demonstrated that exercise performance and cardiac function are relatively well preserved. The risk of developing arrhythmias is low in these patients. For patients well into the adult age range, who may have been treated according to outdated concepts and/or strategies, this may be highly different [1].

The consequence of the impressive advances is that medical professionals involved in the care of patients with congenital heart disease have increasingly started to realise that the focus of care may need to be shifted away from preventing mortality to optimising quality of life. Remarkably, medical advances have not clearly improved health-related quality of life in children so far [2].

Quality of life is an ambiguous concept. Consensus about its definition is lacking. This is reflected in the literature on the subject of quality of life in congenital heart disease, which has shown diverse and sometimes conflicting results [3]. Quality of life is hampered by physical limitations, repeat surgery and the need for ICD implantation. Most, but not all, studies have agreed on the limited role of the type of heart defect on quality of life [3]. This is remarkable, since physical performance and the risk for arrhythmias are clearly related to the type of heart defect [1]. This discrepancy underlines the notion that quality of life has a multifactorial origin [3].

An important aspect that contributes to quality of life is the ability to maintain stable relationships. A study in a large random sample derived from the ConCor registry showed that young adult patients (<40 years old) with congenital heart disease were less likely to be in a relationship compared with the reference group [4].

Positive associations with quality of life have been shown for daily activity levels, academic performance and employment rate. Unfortunately, employment rates are lower for patients with congenital heart disease than for healthy peers. A large study using the ConCor registry has shown that adults with congenital heart disease are more likely to have a lower education level and are unemployed more often [4]. Another Dutch longitudinal study with 20–33 years of follow-up demonstrated impairments in living situation, occupational and educational status, which were related to the severity of the congenital heart defect [5].

Neuropsychological problems, often subtle, have been described extensively for children and adolescents with congenital heart disease. Considering these neuropsychological impairments, limitations in occupational status were to be expected.

On a highly practical level, the study by Sluman et al. in the current issue of the journal aimed to study factors experienced as barriers or facilitators of employment in patients with congenital heart disease [6]. By means of structured interviews with 15 employed congenital heart patients these factors were explored. In 5 of the 15 patients the presence of congenital heart disease had been an important factor in vocational planning. Physical aspects, such as work load, lack of opportunity for recovery (finding the proper balance between work and rest) and relationships with employers were among the most important barriers. Facilitators for employment were less physically demanding work, job autonomy, adequate balance between work and private life and perceived support from colleagues and employer.

The study provides a background to develop effective measures that can be used to help patients with congenital heart disease achieve stable employment-related satisfaction, which relates to quality of life. Ultimately the goal of this type of study is to come up with strategies to empower patients with congenital heart disease. The immense successes of the treatment of these patients should be extended to successful integration of patients with cured but also with chronic congenital heart disease in all aspects of social life, allowing them career choices with as few limitations as possible.
